# Personality-Related Determinants of Physical Activity Among Polish and Spanish Physical Education Students

**DOI:** 10.3389/fpsyg.2021.792195

**Published:** 2022-01-10

**Authors:** Maria Gacek, Grażyna Kosiba, Agnieszka Wojtowicz, Guillermo F. López Sánchez, Jacek Szalewski

**Affiliations:** ^1^Department of Sports Medicine and Human Nutrition, University of Physical Education in Kraków, Kraków, Poland; ^2^Department of Theory and Methodology of Physical Education, University of Physical Education in Kraków, Kraków, Poland; ^3^Department of Psychology, University of Physical Education in Kraków, Kraków, Poland; ^4^Faculty of Health, Education, Medicine and Social Care, School of Medicine, Vision and Eye Research Institute, Anglia Ruskin University, Cambridge, United Kingdom

**Keywords:** physical activity, IPAQ, personality, Big Five model, physical education students

## Abstract

We aimed to analyze personality-related determinants of physical activity among Polish and Spanish physical education (PE) students. The study was conducted among 219 Polish and 280 Spanish PE students, using the International Physical Activity Questionnaire (IPAQ) and the NEO-FFI Personality Inventory. Compared with Spanish PE students, their Polish counterparts are characterized by a higher level of extraversion and conscientiousness and a lower level of neuroticism. The level of total physical activity for all students was 8,697.21 METs, and this value was higher among Polish students. Among Polish and Spanish PE students, the level of total, vigorous, and moderate physical activity increased along with the increase in extraversion, while a decrease occurred along with the increase in neuroticism. The level of each domain of physical activity also increased in line with the intensification of student conscientiousness. In moderation analyses, it was shown that the home country of students may be considered a moderator of the relationship between conscientiousness and total exercise in such a way that the physical activity increased along with the increase in conscientiousness only among the Polish students. In addition, the country is a moderator of the correlation between moderate physical activity and neuroticism (*p* = 0.031), openness (*p* = 0.049), and conscientiousness (*p* = 0.019), with moderate activity only decreasing among Polish students and increasing along with the increase in openness and conscientiousness. Positive correlations among physical activity, extraversion, and conscientiousness, as well as negative ones with neuroticism, were demonstrated among Polish and Spanish students, and also the moderating impact of the country on the correlation between personality-related dimensions and physical activity.

## Introduction

Physical activity is one of the key aspects of a healthy lifestyle, which is the basic determinant of holistically defined health. Physical activity, along with the features of health training, promotes health creation, prevention of chronic diseases, improvement of emotional state, and delaying involutional changes (Rhodes et al., 2017). Within this context, physical activity in many countries, including Poland and Spain, has been implemented, alongside a rational nutrition model, in the canon of basic recommendations for a healthy lifestyle (the WHO pyramid).

A group with a particular level of physical activity is physical education (PE) students who are preparing to become PE teachers. Mandatory and optional physical activity of PE students causes its level to be higher level than in the case of students with different educational profiles (Fagaras et al., 2015; Kosiba et al., 2019b). PE students who are acquiring subject-related competencies to practice the profession of teaching will be involved in the health education of children and youth in their future work. The effectiveness of the work of teachers in the field of health education depends, among others, on their individual commitment to maintaining a healthy lifestyle, which is an important area of physical activity. In this context, it is worth paying attention to publications on the theoretical foundations of promoting and consolidating physical activity in the lifestyle of individuals (Rhodes et al., 2019, 2021; Rhodes and Sui, 2021) and on the relationships among neuroeducation, motivation, and the physical activity of PE students (Baena-Extremera et al., 2021).

Pro-health behaviors among various population groups are conditioned by a wide spectrum of socioeconomic, cultural, and personality-related factors (Birkenhead and Slater, 2015). In studies, the conceptual and empirical significance of individual differences as an important prognostic factor in assessing pro-health behaviors has been demonstrated. In earlier studies among Polish students of PE and other teaching specializations, statistically significant tendencies toward an increase on the scale of pro-health behaviors and the level of physical activity along with the increase in life satisfaction (Kosiba et al., 2016) and with the intensification of some of the features of readiness for change (confidence, passion, and optimism), have been confirmed (Kosiba et al., 2019c). It has also been established that students who rate vital values higher (including fitness and physical strength as well as endurance) undertake physical activity more often (Kosiba et al., 2019a). It has also been confirmed that the level of physical activity increases along with the rise in the sense of generalized self-efficacy (Gacek et al., 2020) and the significance of school PE for the physical activity of students as well as their lifestyle (Carballo-Fazanes et al., 2020).

An interesting area of cognitive research seems to be the assessment of the predictive significance of personality traits included in the Costa and McCrae five-factor model, covering the dimensions of neuroticism, extraversion, openness to experience, agreeableness, and conscientiousness (Costa and McCrae, 1992) toward various areas of lifestyle, including the level of physical activity.

Earlier studies on the relationships between the personality dimensions included in the five-factor model and health behaviors of academic youth concerned, in particular, the personality determinants of eating disorders and alcohol consumption among American students (Martin et al., 2015), pro-health behaviors also among these students (Raynor and Levine, 2009), addiction to physical exercise among French students (Kern, 2010), and the nutritional behaviors of students in Ghana (Intiful et al., 2019) and in Poland and Spain (Gacek et al., 2021). A review of the literature and a meta-analysis of research on the relationships between personality traits and physical activity confirmed the importance of extraversion, neuroticism, and conscientiousness as correlates of physical activity (Rhodes and Smith, 2006; Wilson and Dishman, 2015). In an alternative meta-analysis (Sutin et al., 2016), it was also confirmed that undertaking physical activity was associated with higher conscientiousness, openness and extraversion, and lower neuroticism. In another meta-analysis, correlations were demonstrated between not taking up physical activity and higher neuroticism as well as lower conscientiousness (Allen et al., 2017). In new research, it has been found that moderate exercise is negatively associated with agreeableness and sensitivity to anxiety (Hearon and Harrison, 2021). The relationships of physical activity with personality and character traits in adults were also confirmed by authors from Finland (Karvonen et al., 2020). However, the results of some studies were ambiguous and their authors suggested the validity of conducting further research (Rhodes and Smith, 2006; Martin et al., 2015; Wilson and Dishman, 2015). The ambiguity of results was also related to the importance of potential moderators of personality and physical activity relationships, including gender, age, and culture/country, due to the limited number of subject-related studies (Wilson and Dishman, 2015; Allen et al., 2017).

In this context, research has been undertaken regarding the predictive role of personality traits included in the five-factor model related to the level of physical activity among Polish and Spanish PE students, who, according to the core curriculum for PE, will be the main promoters of a healthy lifestyle in their future professional work. Assuming that health-promoting behaviors, including physical activity, are key determinants of health, and personality traits play an important role in their formation, and furthermore, that PE students will take up work and be involved in the health education of school youth, research on personality determinants has been executed concerning the level of physical activity among Polish and Spanish PE students.

The aim of the study was to analyze the intensity of personality traits included in the five-factor model (neuroticism, extraversion, openness to experience, agreeableness, and conscientiousness), the level of physical activity [according to the International Physical Activity Questionnaire (IPAQ)], and the relationship between these variables, as well as the analysis of the moderating importance of the home country of students among Polish and Spanish PE students in relation to the examined correlations. Two research questions were posed as follows: (i) What are the correlations between the intensity of personality traits and the level of physical activity of PE students?; and (ii) Does the country of residence moderate the personality and physical activity relationships of Polish and Spanish PE students? Two research hypotheses were subjected to empirical verification as follows: (i) personality traits according to the Big Five model determine the level of physical activity among PE students, and (ii) country of origin moderates the correlation between personality and physical activity of Polish and Spanish PE students.

## Materials and Methods

### Participants

This research was conducted in 2017–2019 among 499 second- and third-year BA (undergraduate) students, aged 18–35 years (*M* = 21.65 and SD = 2.42). This study included 219 Polish students (from the University of Physical Education in Krakow and Wrocław) and 280 Spanish students (from the University in Murcia and Granada) of PE. The study group included 189 women (37.88%) and 310 men (62.12%). The basic criterion for inclusion in the group was studying PE (second- and third- year of BA studies). The greatest percentage of Polish students came from urban (35.2%) and rural areas (32.0%), less often from medium-sized (19.5%) and small towns (13.3%). A similar percentage of students from Spain came from urban areas (27.1%), small (26.1%), as well as medium-sized cities (24.6%), and from the countryside (22.2%). Questionnaires were conducted in Poland and Spain at the same time (in spring). The trial was carried out in an auditory fashion (paper and pencil) by trained persons.

### Measures

In this research, the diagnostic survey method and two research tools, namely, the IPAQ (Polish: Biernat et al., 2007; Spanish: Roman-Viñas et al., 2010) and the NEO-FFI Personality Inventory (Costa and McCrae, 1992; Polish: Zawadzki et al., 1998; Spanish: Costa and McCrae, 1999), were used.

Based on the short version of the IPAQ, the level of physical activity was assessed, including four categories as follows: vigorous activity (above 1,500 or 3,000 MET-min/week) and moderate physical activity (600–1,500 or 600–3,000 MET-min/week) as well as walking (below 600 MET-min/week) and sitting (Biernat et al., 2007).

The NEO-FFI questionnaire, referring to the popular Big Five model (factor analysis that has been applied to personality survey data that revealed semantic associations), was used to assess personality traits (Costa and McCrae, 1992). The questionnaire measures the level of five personality traits as follows: neuroticism (sensitive/nervous vs. resilient/confident), extraversion (outgoing/energetic vs. solitary/reserved), openness to experience (inventive/curious vs. consistent/cautious), agreeableness (friendly/compassionate vs. challenging/callous), and conscientiousness (efficient/organized vs. extravagant/careless). NEO-FFI includes a total of 60 self-report items, the truth of which is assessed by the respondent on a five-point scale, in which, 1 means “I strongly disagree,” 2–“I disagree,” 3–“I have no opinion,” 4–“I agree,” 5–“I strongly agree.” In the Polish version, the internal reliability of the tool is satisfactory with Cronbach’s alpha from 0.68 to 0.82 (Zawadzki et al., 1998), similar to the Spanish version with Cronbach’s alpha from 0.71 to 0.82 (Manga et al., 2004).

This research was carried out as part of a scientific project entitled “Health behaviors among students of teaching faculties in selected European countries within the context of their future professional role as health educators–analysis of selected formal and legal, socio-cultural and psychological determinants” (the University of Physical Education in Kraków, project number: 1136/BS/INS/2017), conducted in accordance with the principles of the 1964 Declaration of Helsinki after obtaining the informed consent of subjects for participation.

### Statistical Analysis

The IBM SPSS 21 program (IBM, Armonk, NY, United States) was used for all statistical calculations with the J. T. Newsom macro for conducting moderation analysis. Raw scores were used for statistical analysis. Basic statistics of the studied variables were calculated (means and SDs). *T*-test with a separate estimation of variance was used for comparisons between Polish and Spanish students, Pearson’s correlation analysis was used to determine the relationships between variables, while moderation analysis with simple slopes (the regression of the outcome *y* on the predictor *x* at a specific value of the moderator *z*) was applied to determine the differences in the relationships between personality traits and the physical activity of the studied PE students. The significance level of *p* = 0.05 was adopted. Due to the simple slope results, the interaction effects (moderation) with *p* < 0.052 were also reported.

## Results

In Table 1, basic descriptive statistics of the tested variables are presented, and comparisons between PE students from Poland and Spain are shown. Polish students obtained higher average results than Spanish students in the categories of extraversion and conscientiousness, higher values for vigorous effort indicators, walking, moderate exercise indices, and general IPAQ index, but lower average results in the category of neuroticism. There were no statistically significant differences between groups in the level of agreeableness, openness, and sitting.

**TABLE 1:** Personality traits (NEO-FFI) and the level of physical activity (IPAQ) among Polish and Spanish physical education (PE) students (means, SDs, and analysis of differences tests).

	All		Country				*p*-value
			Mean		SD		
	Mean	SD	P	S	P	S	
Neuroticism	33.34	6.26	32.58	33.93	8.57	3.43	0.029
Extraversion	40.47	5.71	43.34	38.23	6.04	4.26	<0.001
Openness	37.15	4.36	36.92	37.34	5.48	3.22	0.323
Agreeableness	39.69	4.58	39.84	39.58	5.52	3.68	0.534
Conscientiousness	37.89	7.11	43.79	33.28	6.46	2.98	<0.001
IPAQ vigorous	4,036.51	5,461.66	4,809.27	3,506.36	7,983.94	2,455.10	0.037
IPAQ moderate	1,908.93	2,333.63	2,367.80	1,591.39	2,974.52	1,694.61	0.003
IPAQ walking	2,917.82	3,484.79	4,273.59	1,916.46	4,309.67	2,254.44	<0.001
IPAQ sitting	266.69	150.55	249.72	278.05	146.80	152.26	0.065
IPAQ total	8,697.21	8,285.49	11,376.87	6,871.93	11,510.16	4,163.82	<0.001

*SD, standard deviation; P, Poland; S, Spain.*

In the analysis of relationships between personality traits and the level of physical activity for the whole group, it was shown that as the level of neuroticism increased, the level of vigorous, moderate, and total physical activity decreased, while the level of sitting increased. At the same time, the higher the level of extraversion, the higher were the levels of vigorous, moderate, and total physical activity as well as walking, while the level of sitting was lower. It was also shown that as conscientiousness increased, the level of each of the domains of physical activity increased while the level of sitting decreased (Table 2).

**TABLE 2:** Personality traits (NEO-FFI) and the level of physical activity (IPAQ) among Polish and Spanish PE students (Pearson’s correlation coefficient analysis).

IPAQ domains		Neuroticism			Extraversion			Openness			Agreeableness			Conscientiousness		
		All	P	S	All	P	S	All	P	S	All	P	S	All	P	S
IPAQ vig	r	−0.101	−0.107	0.027	0.170	0.160	0.086	−0.010	−0.008	0.003	0.016	0.051	−0.097	0.167	0.154	−0.029
	*p*	0.036	0.156	0.667	0.001	0.034	0.168	0.835	0.920	0.958	0.736	0.497	0.118	0.001	0.041	0.646
	*n*	435	177	258	435	177	258	435	177	258	435	177	258	435	177	258
IPAQ mod	r	−0.176	−0.231	0.045	0.224	0.235	0.059	0.053	0.130	−0.076	0.070	0.094	0.035	0.205	0.203	−0.068
	*p*	0.001	0.003	0.488	0.001	0.002	0.365	0.289	0.096	0.244	0.160	0.232	0.594	0.001	0.009	0.297
	*n*	401	164	237	401	164	237	401	164	237	401	164	237	401	164	237
IPAQ walking	r	−0.045	−0.011	−0.003	0.142	−0.002	0.013	−0.046	−0.027	−0.004	−0.093	−0.141	−0.028	0.274	0.071	−0.044
	*p*	0.360	0.879	0.967	0.004	0.978	0.839	0.351	0.720	0.951	0.057	0.060	0.671	0.001	0.346	0.494
	*n*	419	178	241	419	178	241	419	178	241	419	178	241	419	178	241
IPAQ sitting	r	0.100	0.157	0.011	−0.167	−0.123	−0.166	−0.006	−0.007	−0.013	0.043	−0.030	0.119	−0.095	−0.024	−0.072
	*p*	0.045	0.048	0.868	0.001	0.123	0.010	0.903	0.930	0.838	0.390	0.711	0.065	0.058	0.764	0.269
	*n*	399	160	239	399	160	239	399	160	239	399	160	239	399	160	239
IPAQ total	r	−0.172	−0.171	−0.001	0.248	0.193	0.063	−0.017	0.005	−0.059	0.001	0.019	−0.049	0.290	0.191	−0.047
	*p*	0.001	0.043	0.994	0.001	0.022	0.367	0.745	0.948	0.395	0.986	0.827	0.48	0.001	0.023	0.501
	*n*	348	141	207	348	141	207	348	141	207	348	141	207	348	141	207

*Vig, vigorous; Mod, moderate; r, Pearson’s correlation coefficient; p, significance; P, Poland; S, Spain.*

From the statistical analyses, it may be deduced that the home country of students can be considered a moderator of the relationship between conscientiousness and the total level of physical activity (*p* = 0.051). Among Polish PE students, the level of total physical activity increased along with the increase in conscientiousness, while among Spanish students, this relationship was insignificant and slightly negative (Table 3).

**TABLE 3:** Moderation analysis–moderating variable: country; dependent variable: IPAQ total.

Dependent variable	Moderator	Independent variable	β	SE	*t*	*p* interaction	Slopes
IPAQ Total	Country	Neuroticism	0.07	0.06	1.23	0.219	β_P_ = −0.17 (*p* = 0.004) β_S_ < 0.01 (*p* = 0.997)
		Extraversion	−0.11	0.07	−1.64	0.102	β_P_ = 0.24 (*p* = 0.001) β_S_ = 0.04 (*p* = 0.633)
		Openness	−0.03	0.06	−0.41	0.679	β_P_ = 0.01 (*p* = 0.926) β_S_ = −0.04 (*p* = 0.658)
		Agreeableness	−0.03	0.07	−0.48	0.628	β_P_ = 0.02 (*p* = 0.753) β_S_ = −0.03 (*p* = 0.713)
		Conscientiousness	−0.15	0.08	−1.96	0.051	β_P_ = 0.29 (*p* = 0.001) β_S_ = −0.05 (*p* = 0.722)

*β, standardized coefficient beta; SE, standard error; p, significance; P, Poland; S, Spain.*

It has been shown that the country is a moderator of the correlation between moderate physical activity and neuroticism (*p* = 0.031), openness (*p* = 0.049), and conscientiousness (*p* = 0.019) (Table 4). Among Polish students, the higher the level of neuroticism, the lower was the level of moderate physical activity. Moreover, the higher the level of openness and conscientiousness, the greater the level of moderate physical activity. Among Spanish students, these relationships did not reach statistical significance and all were opposite (Table 4).

**TABLE 4:** Moderation analysis–moderating variable: country; dependent variable: IPAQ moderate.

Dependent variable	Moderator	Independent variable	β	SE	*t*	*P* interaction	Slopes
IPAQ Moderate	Country	Neuroticism	0.12	0.05	2.16	0.031	β_P_ = −0.21 (*p* < 0.001) β_S_ = 0.06 (*p* = 0.603)
		Extraversion	−0.13	0.06	−1.94	0.053	β_P_ = 0.27 (*p* < 0.001) β_S_ = 0.06 (*p* = 0.498)
		Openness	−0.12	0.06	−1.97	0.049	β_P_ = 0.13 (*p* = 0.032) β_S_ = −0.07 (*p* = 0.389)
		Agreeableness	−0.04	0.06	−0.69	0.488	β_P_ = 0.10 (*p* = 0.123) β_S_ = 0.03 (*p* = 0.694)
		Conscientiousness	−0.18	0.07	−2.35	0.019	β_P_ = 0.28 (*p* = 0.001) β_S_ = −0.11 (*p* = 0.437)

*β, standardized coefficient beta; SE, standard error; p, significance; P, Poland; S, Spain.*

In the analyses, it was not shown that home country was a moderator of correlations between personality and vigorous physical activity or the “walking” domain of physical activity (IPAQ walking).

In the conducted analyses, it was not demonstrated that the country was a moderator of the correlation between personality and sitting.

## Discussion

Among Polish and Spanish students of PE, the differentiation of selected personality traits was demonstrated according to the Big Five model. A high level of physical activity was also noted, with an indication of higher results in the domain of vigorous and moderate exercise and walking among Polish students. In addition, statistically significant relationships between the intensity of some personality traits and the level of total physical activity and its individual domains specified in the IPAQ have been demonstrated. It has also been shown that the relationships of some dimensions of personality with the level of physical activity and its individual domains specified in the IPAQ were moderated by the country of origin of PE students (Poland vs. Spain).

Differentiation in the level of individual personality traits included in the Costa and McCrae five-factor model for Polish and Spanish PE students, with an indication of the high intensity of extraversion and the low intensity of neuroticism, may be associated with the specificity of the field of study focused on physical activity, which can meet the high demand for stimulation (in people with high extraversion) and improve their emotional state. In other studies among teachers in Serbia emphasized a high level of self-efficacy, the main predictors of which were two personality dimensions from the so-called Big Five, i.e., conscientiousness and openness (Djigić et al., 2014). Their high level was also described–with reference to Polish and Spanish PE students–in future teachers.

The demonstrated high level of physical activity among Polish and Spanish PE students, including domination of the domain of vigorous efforts, is clearly associated with the profile of education of the studied academic youth. The results obtained in this respect correspond to the results of studies by other authors, which confirm a significantly higher level of physical activity among PE students than those with a different educational profile (Fagaras et al., 2015; Antoniazzi et al., 2018; Kosiba et al., 2019b). In other studies, a higher level of physical activity has been demonstrated among Spanish students compared with those from Poland (Lopez-Sanchez et al., 2019).

In the discussed research conducted by the authors, statistically significant correlations have been shown between some personality traits included in the five-factor model and the level of physical activity as well as its individual domains (according to IPAQ) among Polish and Spanish PE students. It was found that with the increase in the level of neuroticism associated with emotional liability, low self-esteem, and high sensitivity, the level of total physical activity and its domains in the field of vigorous and moderate physical activity significantly decreased. In contrast, the amount of time spent passively (sitting) increased along with the increase in the level of neuroticism. In turn, with the increase in the level of extraversion, negatively correlated with neuroticism, associated with sociability, assertiveness, vigor, and search for sensations/emotions, the level of total physical activity, its domains in the field of vigorous and moderate efforts and walking increased. It has also been shown that with the increase in conscientiousness associated with obligation, discipline, and pursuit of specific goals, the level of each of the physical activity domains also increased. A high level of conscientiousness, related to responsibility, also for the health of individuals (in accordance with the principle of the subjectivity of health), is conducive to undertaking behaviors that increase health potential, the key form of which is an active lifestyle associated with undertaking physical activity. Therefore, it may be concluded that among the personality traits included in the five-factor model, a high level of extraversion and conscientiousness and a low level of neuroticism among PE students have a positive significance for the high physical activity level of Polish and Spanish PE students. It should be emphasized in this study that Polish and Spanish PE students obtained high results in the category of extraversion, agreeableness, conscientiousness, and openness and low results in the field of neuroticism, which is a positive psychological basis for their high physical activity.

The regularities in the discussed research regarding personality determinants of the level of physical activity among Polish and Spanish PE students are explained in the characteristics of the analyzed personality dimensions, having theoretical foundations, they are theoretically reliable and refer to the results of research by other authors on the psychological determinants of pro-health behaviors among various population groups, including students. In studies conducted among American students, it has been confirmed that extraversion and conscientiousness are strong predictors of their pro-health behavior. Students with high levels of conscientiousness declared a more healthy lifestyle, including physical activity (Raynor and Levine, 2009). In other studies on the importance of conscientiousness and extraversion (the dimensions of the Big Five) in undertaking physical activity, it has been shown that extraversion was conducive to performing moderate physical, while conscientiousness was conducive to vigorous physical activity (De Bruijn et al., 2009). In research on the relationship between personality, physical activity, and muscle strength among adults, it has been demonstrated that neuroticism was negatively while extroversion was positively correlated with muscle strength that may be derived from physical activity (Tolea et al., 2012). A systematic review and meta-analysis of studies on personality determinants of physical activity confirmed the positive significance of extraversion and conscientiousness and the negative significance of neuroticism as factors determining the level of physical activity (Wilson and Dishman, 2015; Sutin et al., 2016; Allen et al., 2017).

From the results obtained in the research of authors on the moderating impact of the country of origin of examined PE students (Poland vs. Spain) with reference to the studied relationships, it can be stated that the country of origin is a moderator of some of the analyzed relationships of personality traits and the level of physical activity among students. It has been shown that the home country of students may be considered as a moderator of the relationship between conscientiousness and total physical activity, while along with the increase in the level of conscientiousness, the level of physical activity only increased among Polish students. It has also been shown that the country is a moderator of the relationship between moderate physical activity and neuroticism and openness and conscientiousness, while the level of moderate physical activity decreased with the increase in neuroticism and increased with the increase of openness and conscientiousness only in Polish students. In addition, it was found that the country was not a moderator of relationships between personality traits and the amount of time spent on vigorous activity, walking, and sitting. Personality is considered to be an intercultural trait, and therefore, the relationship between physical activity and personality traits in different cultures should be stable. A systematic review and meta-analysis of studies on personality determinants of physical activity did not allow for unambiguous identification of potential moderators regarding personality and physical activity relationships, including gender, age, and culture/country (mainly due to the small number of studies) (Rhodes and Smith, 2006). The research also allowed to confirm the moderating significance of the geographical/cultural factor with regard to the relationship between the sense of self-efficacy and physical activity of PE students (Gacek et al., 2020). There are reports in the literature suggesting alleviation of the relationship between physical activity and neuroticism through the interaction of gender and geographical region, with the results of the study not explicit (Wilson and Dishman, 2015). Therefore, numerous authors (Wilson and Dishman, 2015) pointed to the need to continue international research on factors conditioning and moderating the level of physical activity, taking various variables into account, including gender and geographical region (nation) as well as psychological (personality) and environmental factors, into which the authors of the presented work attempted to incorporate this suggestion.

Concluding, the limitations and strengths of the study should be indicated. The limitations mainly result from the nature of the research (questionnaires and self-report tools); therefore, the collected data are of declarative nature. There are also limitations related to not considering socio-economic factors in the analyses. Subsequent research, taking into account a wider spectrum of individual and environmental factors, may contribute to the development of a complex model of determinants related to the physical activity of the level of students. The strength of the study is its interdisciplinary nature (health promotion and health psychology), as well as the selection of students from different cultures preparing for the profession of a PE teacher and carrying out school health education.

Becoming acquainted with the personality determinants regarding the physical activity of future teachers may favor the individualization of interactions related to health promotion, which further determines the applicative meaning of work. At the same time, it is necessary to point out the key importance of the personal involvement of a teacher in the implementation of a healthy lifestyle and the need to increase the effectiveness of preparing PE students for the role of health educators, taking their individual predispositions into account.

## Conclusion

(1) Among the personality traits included in the five-factor model, Polish PE students obtained higher scores for the categories of extraversion and conscientiousness and lower values for the category of neuroticism than Spanish students.
(2) Among the surveyed students of PE, a high level of total physical activity was described, with Polish students achieving higher results in the domain of vigorous and moderate exercise and walking than Spanish students.
(3) Among the Polish and Spanish PE students, statistically significant relationships were found between the intensity of some personality traits and the level of total physical activity as well as its individual domains (according to the IPAQ), with an indication of an increase in the level of physical activity along with an increase in extraversion and conscientiousness and a decrease in neuroticism.
(4) Among Polish and Spanish PE students, the moderating influence of home country on the relationships of some dimensions of personality with the level of total physical activity and its domains (according to the IPAQ) has been demonstrated, with an indication of the increase in the level of total physical activity along with the intensification of conscientiousness, also an increase in moderate activity along with an increase in openness and conscientiousness, and a decrease in neuroticism only among Polish students.

## Data Availability Statement

The raw data supporting the conclusions of this article will be made available by the authors, without undue reservation.

## Ethics Statement

The studies involving human participants were reviewed and approved by the University of Physical Education in Kraków Institutional Review Board (NN/602-33/17). The patients/participants provided their written informed consent to participate in this study.

## Author Contributions

MG, GK, and AW: study design. GK, MG, AW, JS, and GL: data collection. MG and AW: statistical analysis. MG, GK, AW, and GL: manuscript preparation. GK: project administration. All authors have read and agreed to the published version of the manuscript.

## Conflict of Interest

The authors declare that the research was conducted in the absence of any commercial or financial relationships that could be construed as a potential conflict of interest.

## Publisher’s Note

All claims expressed in this article are solely those of the authors and do not necessarily represent those of their affiliated organizations, or those of the publisher, the editors and the reviewers. Any product that may be evaluated in this article, or claim that may be made by its manufacturer, is not guaranteed or endorsed by the publisher.
